# Validation of the Retinopathy of Prematurity Activity Scale (ROP-ActS) using retrospective clinical data

**DOI:** 10.1111/aos.14532

**Published:** 2020-06-26

**Authors:** Aldina Pivodic, Staffan Nilsson, Andreas Stahl, Lois E.H. Smith, Ann Hellström

**Affiliations:** 1 Department of Ophthalmology, Institute of Neuroscience and Physiology, Sahlgrenska Academy, University of Gothenburg, Gothenburg, Sweden; 2 Statistiska konsultgruppen, Gothenburg, Sweden; 3 Mathematical Sciences, Chalmers University of Technology, Gothenburg, Sweden; 4 Institute of Biomedicine, University of Gothenburg, Gothenburg, Sweden; 5 Department of Ophthalmology, University Medical Center Greifswald, Germany; 6 Department of Ophthalmology, Boston Children’s Hospital, Harvard Medical School, Boston, MA, USA

**Keywords:** retinopathy of prematurity, ROP activity scale, ROP-ActS, ROP stage, ROP zone

## Abstract

**Purpose:**

The International Neonatal Consortium recently published a proposed retinopathy of prematurity (ROP) activity scale intended for use in clinical trials after validation. The aim of this study was to validate the ROP activity scale (ROP-ActS) in a ROP screened cohort with protocol based collected data by evaluating the ability of the ROP-Act scores to predict ROP treatment. In addition, we aimed to evaluate the scale’s sensitivity characteristic of disease severity by studying association with gestational age (GA) in comparison with conventionally used ROP stage and zone.

**Methods:**

A cohort of 535 preterm infants with 3324 ROP examinations with an end-point of ROP treatment or end of screening in Gothenburg, Sweden, was included. Median GA was 28.1 weeks, 47.5% were girls, and 74 (13.8%) infants were treated for ROP. The validation was performed by estimating probabilities for ROP treatment, and by applying logistic and linear regression.

**Results:**

The original ROP-ActS was overall well-ordered with respect to ability to predict ROP treatment but could be improved by re-ordering score 3 (zone II stage 1) and 5 (zone III stage 3) based on our clinical cohort data. The modified ROP-ActS was superior to ROP stage and zone in the prediction analysis of ROP treatment. Modified ROP-ActS was more strongly related to GA than currently used ROP stage, but not zone.

**Conclusion:**

In the studied cohort, the modified ROP-ActS could better predict ROP treatment compared to ROP stage and zone. Retinopathy of Prematurity Activity Scale (ROP-ActS) had a superior sensitivity characteristic studied through association to GA than conventionally used ROP stage.

## Introduction

The International Neonatal Consortium (INC) is focused on developing objective descriptors of all stages of retinopathy of prematurity (ROP) to discriminate effects of prevention and treatment. Current studies apply the parameters stage, location (zone I-III), extent of proliferative disease and preplus/plus disease of the International Classification of Retinopathy of Prematurity (ICROP) for classification (ICROP, 2005). The decision to treat severe ROP is based on a defined combination of these variables (type 1 ROP [treatment-requiring] and type 2 ROP [not requiring treatment]). Clinical studies reporting ROP severity most commonly use type or stage only (Stahl et al., 2018; Stahl et al., 2019; Tu et al., 2019).

Regulatory authorities asked members of the INC to develop an ROP activity scale (ROP-ActS) that would improve sensitivity of disease severity (Smith et al., 2019). The more sensitive the studied measure is, the better the ability to discriminate between treatments, and the higher the statistical power in clinical trials. The developed ROP-ActS is based on a scale which included all theoretically possible combinations of the three currently used ICROP determinants defining ROP severity (zone, stage and presence of plus disease) (ICROP, 2005). These 23 combinations (range 0 [no ROP] to 22 [stage 5 ROP]) were originally ranked by ‘severity’ based on the clinical judgement of nine ROP experts (Smith et al., 2019). Validation studies on a cohort with short-term, long-term and safety outcomes based on well documented, prospectively or retrospectively collected data were considered necessary in the original publication of the scale (Smith et al., 2019). Until now, the ROP-ActS has not been evaluated on a clinical cohort.

The primary aim of the current study was to validate the ROP-ActS using data from all prematurely born infants that were screened for ROP during 2013–2018 at Sahlgrenska University Hospital in Gothenburg, Sweden. Secondly, it was to study the order of severity of the various scale components to predict ROP treatment and to modify the scale if needed. Additionally, we wished to evaluate if the initial ROP-ActS scale could predict ROP treatment and its association to GA compared to currently used ROP stages and zones.

## Materials and Methods

The study was approved by the ethical review board in Sweden Dnr 2019-02321.

### Study procedures

Gestational age (GA) was estimated from the postmenstrual week 18 fetal ultrasound. For infants born at GA ≥ 24 weeks, a standard deviation score (SDS) showing difference from expected reference birthweight (BWSDS) was calculated, based on GA at birth, BW and sex (Niklasson & Albertsson-Wikland, 2008). All data were reported at the study site according to a standardized ROP protocol. Five experienced ophthalmologists performed the ROP examinations. Retinopathy of Prematurity (ROP) stage, zone and status of plus disease were defined according to the International Classification of Retinopathy of Prematurity (ICROP, 2005). Those three variables were also used for the definition of the ROP-ActS (Table S1). The worst case of left and right eye was analysed.

### Study outcome

Retinopathy of prematurity (ROP) treatment, following the Early Treatment of Retinopathy of Prematurity (ETROP) criteria (ETROP, 2003), is the study outcome. For infants treated for ROP, data up to the date of the first treatment were used. For all other infants, data from all ROP screening examinations were included in the analyses.

### Study population used for validation of the ROP Activity Scale (ROP-ActS)

Informed consent from the parents/guardians was obtained to collect ROP screening data for the Swedish National Registry for Retinopathy of Prematurity (SWEDROP) to be used for clinical care improvement.

All 535 infants screened for ROP between 1 January 2013 and 31 December 2018 at the paediatric ophthalmology department at the Queen Silvia Children’s Hospital in Gothenburg, Sweden, were included in this study. There were 3898 screening examinations; 419 (10.7%) post-treatment examinations were excluded for infants treated for ROP, leaving 3479 (89.3%) available for consideration. Of those, 3324 (95.5%) had the reported data required for evaluation of the ROP-ActS. Reasons for missing data were retrieved from the medical records. Seventy-one (2.0%) examinations, occurring most often at the start of the screening, were excluded because examiners were unable to assess very immature retinas and/or for insufficient dilatation of the pupil. Additionally, 10 (0.3%) visits were excluded because of difficult or incomplete examinations (*n* = 4), unstable very sick infant (*n* = 4), regression reported without specified detailed data (*n* = 1) and patient examined at another site *n* = 1). The ROP-ActS was not evaluated for 74 (2.1%) visits that occurred on the day of the first ROP treatment. Therefore, data from those 74 examinations were used to define the status and timing of ROP treatment.

### Statistical analysis

Categorical variables were described by number and percentage, and continuous variables by mean, standard deviation (SD), median, minimum and maximum.

The logical partial order restrictions based on increasing severity: ROP stages 1–3; zone III–I; no plus to plus disease, were checked and observed fulfilled for the ROP-ActS (Appendix S1). The order of the scores in the activity scale according to the severity of the disease was evaluated by calculating the percentage with 95% confidence intervals (CI) of infants with incident ROP treatment among infants ever experiencing a certain score. The percentages were numerically compared and graphically presented in bar charts. The modified ROP-ActS was suggested based on the outcome from these analyses of the scale’s correspondence to the severity of the disease.

The ability of the most severe value before 10 weeks of postnatal age of the original, modified ROP-ActS, ROP stage and zone to predict ROP treatment (end-point) was analysed using univariable and multivariable logistic regression, with risks studied linearly increasing by one step increase. The multivariable analysis was performed to study whether modified ROP-ActS, ROP stage and zone would remain significant predictors when adjusted for each other, and when adjusted for GA, weight and sex. Odds ratio (OR) with 95% CI and area under receiver operating characteristic (ROC) curve were presented.

In order to examine the preference of selecting either of the original, modified ROP-ActS, ROP stage or zone as exposure variable in a study, the association between the most severe values of the four variables before week 10 of postnatal age and gestational age was analysed using univariable and multivariable linear regression. The associations were studied among all infants and among those experiencing any ROP up to 10 weeks of postnatal age, studying linear effects between the four independent variables and GA. This exercise was performed in order to evaluate whether modified ROP-ActS would be a more sensitive variable regarding severity of the disease, and therefore stronger correlated to GA than ROP stage and zone. Beta estimates with 95% CI, and *R*^2^ were presented.

All tests were two-tailed and conducted at 0.05 significance level. All analyses were performed using SAS software version 9.4 (SAS Institute Inc., Cary, NC, USA).

## Results

### Study population and examinations

Table 1 presents descriptive data for the overall study population and for infants with and without ROP treatment. Among 535 infants included, 254 (47.5%) were girls, mean birthweight was 1068 (SD 358) grams and median GA 28.1 (range 22.4–34.4) weeks. Mean BWSDS was −1.16 (SD 1.53) among infants born with a GA ≥24 weeks. Mean number of screening examinations with available ROP-ActS score data was higher for treated than for non-treated infants (mean 7.7 [range 2–24] versus mean 6.0 [range 1 −30], respectively).

### Observed ROP stage, plus disease, zone and ROP Activity Scale (ROP-ActS)

In this study population, 74 (13.8%) infants were treated for ROP at least once (24 born at GA 22–23 weeks [70.6% of that group], 31 born at GA 24–25 weeks [36.0% of that group] and 19 born at GA 26–27 weeks [14.4% of that group]); no ROP treatment was required for any infant born with GA ≥28 weeks. Maximum ROP stage observed was stage 3 in 107 (20.0%) infants, stage 2 in 89 (16.6%), stage 1 in 42 (7.9%), leaving 297 (55.5%) screened infants with no diagnosed ROP. Among infants with diagnosed ROP, the most central zone was zone I in 2 infants only, zone II in 166 (69.7%) and zone III in 70 (29.4%). 60 (25.2%) were diagnosed with plus disease, 1 with stage 2 and 59 with stage 3 ROP, Table 1.

For the majority of infants (466 [87.1%]), the first ROP examination score was 0 (no ROP); 9 (1.7%) had score 1 (zone III stage 1); 8 (1.5%) had score 2 (zone III stage 2); 27 (5.0%) had score 3 (zone II stage 1); 23 (4.3%) had score 7 (zone II stage 2) observed at first screening. At first examination, one infant had score 5 (zone III stage 3) and one infant had score 8 (zone II stage 3). The following scores were not observed in this cohort (and may in part be physiologically unlikely to occur): scores 4 (zone III stage 1+), 6 (zone III stage 2+), 10 (zone I stage 1), 11 (zone II stage 1+), 15 (zone I stage 1+), 17 (zone I stage 2+) and 18 (zone I stage 3+). Other scores that are clinically highly relevant but were also not seen in this cohort were scores 19–22 (AP-ROP, stage 4a, 4b and 5).

Distribution of longitudinal values for ROP-ActS and ROP stages and zones for different gestational weeks is presented in Figs S1, S2 and S3.

### Severity order of the ROP Activity Scale (ROP-ActS) tested against ROP treatment

**Fig. 1.**
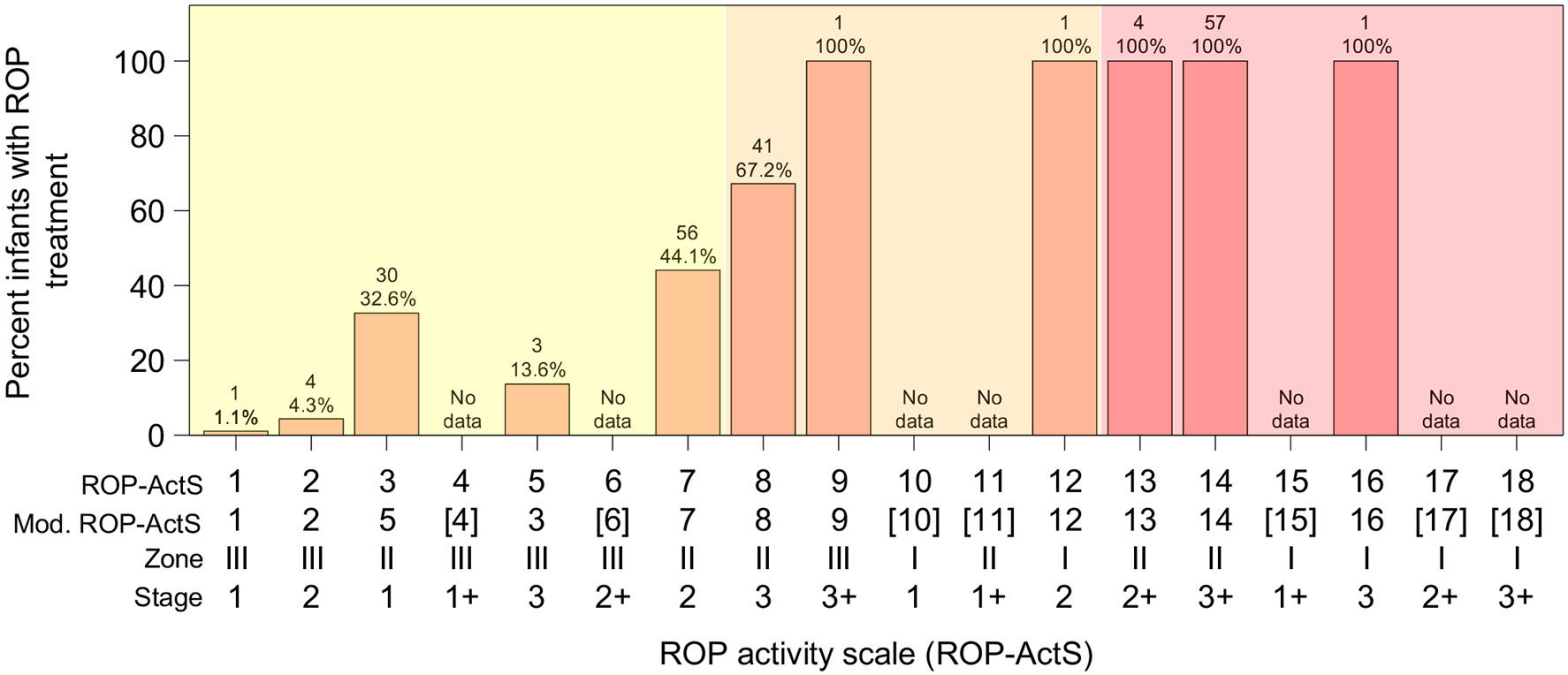
Incidence of ROP treatment for infants ever experiencing ROP Activity Scale (ROP-ActS) scores 1–18. This figure is presenting risk for ROP treatment for infants ever experiencing a certain score, implying that the same infant might be represented in more than one score.

The ROP-ActS fulfilled the logical partial order restrictions based on increasing severity of ROP stage, zone and plus disease (Appendix S1). The proportions of infants with ROP treatment among those with each ROP-ActS score are presented in Fig. 1 (for ROP-ActS scores 1–18). The largest relative numerical increase in the incidence of ROP treatment between two adjacent ROP-ActS scores was seen for score 3 (zone II stage 1) versus score 2 (zone III stage 2), 32.6% (95% CI 23.2%–43.2%) versus 4.3% (95% CI 1.2%–10.8%), respectively. Numerical decrease in the incidence of ROP treatment for an increased ROP-ActS score was observed for score 5 (zone III stage 3 versus score 3 (zone II stage 1), 13.6% (95% CI 2.9%–34.9%) versus 32.6% (95% CI 23.2%–43.2%), respectively. For all other adjacent comparisons, where data were available, equal or numerically higher incidences were found for higher scores.

### The modified ROP Activity Scale (ROP-ActS)

Based on the results from the analyses of correspondence to the severity of the studied outcome, scores 3 and 5 were switched with each other, resulting in the modified ROP-ActS score 3 representing zone III stage 3 and score 5 representing zone II stage 1. All other ROP-ActS scores in the modified version were left unchanged, Fig. 1 and Table S1.

### Prediction ability of the modified ROP Activity Scale (ROP-ActS) versus ROP stage and zone

All four variables, maximum value of the original and modified ROP-ActS, as well as most severe ROP stage and zone, up to postnatal week 10, were significant predictors for ROP treatment. The observed areas under the ROC curves were for the modified ROP-ActS scale 0.82 (95% CI 0.76–0.87), for the original ROP-ActS 0.81 (95% CI 0.76–0.87), followed by ROP zone 0.81 (95% CI 0.76–0.86) and ROP stage 0.79 (95% CI 0.73–0.84), Table 2. The same conclusions were drawn after adjustment for GA, birthweight and sex. The modified ROP-ActS had significantly superior predictive ability compared to stage and zone studied in separate multivariable logistic regression models.

### Association between GA and modified ROP Activity Scale (ROP-ActS) compared to ROP stage and zone

Maximum value of the original and modified ROP-ActS, and most severe ROP stage and zone, up to postnatal week 10, were all significantly associated to GA, analysed on all infants. The highest *R*^2^ of 0.26 was estimated for the modified ROP-ActS, followed by ROP zone 0.25, original ROP-ActS 0.23 and ROP stage 0.17 (Table 3). There was no significant correlation between ROP stage and GA when analysed excluding infants with no ROP. Modified ROP-ActS showed strengthened negative correlation and was superior to ROP stage when studied together in a multivariable model against GA, both including all infants and those with any reported ROP up to postnatal week 10. However, zone showed to be superior to modified ROP-ActS in the association to GA among infants with any ROP.

## Discussion

The current study found that the re-ordering of the theoretically developed ROP activity scale (ROP-ActS) would allow better correspondence to the risk for developing ROP that needed treatment in this cohort. The modified ROP-ActS could predict the studied outcome when applied retrospectively to a large clinical data set, better than the conventionally used both ROP stage and zone when evaluated statistically. The modified scale’s sensitivity characteristic, studied through associations to GA, was shown to be superior compared to ROP stage, but not to ROP zone.

As improved healthcare worldwide increases the number of extremely premature infants at high risk for severe ROP requiring treatment (Holmstrom et al., 2019), there is a need to develop new preventative and safe treatments for proliferative disease (”; ’The MegaDonnaMega trial’; ’The ROPROP trial’; Stahl et al., 2019). In order to have sensitive ROP measures in clinical trials, a theoretical ROP-ActS taking into account disease stage, zone and presence of plus disease was developed (Smith et al., 2019). The more sensitive the studied measure, the better is the ability to discriminate between treatments. This characteristic could be shown in our study by evaluating association between GA and the modified ROP-ActS that was found to be superior to ROP stage in those analyses. However, ROP zone showed even better characteristics in a subgroup of infants with any ROP up to 10 weeks postnatal age. One explanation for this finding may be the close relationship between degree of immaturity and distance of retinal vascular growth from the optic disc towards the periphery (Hughes, Yang, & Chan-Ling, 2000). But, zone was not superior to modified ROP-ActS when studied on all infants, possibly due to a non-linear relation between zone and GA. Better discriminative ability implies higher statistical power in clinical trials which might be further increased by improving the scale’s length of the intervals between the scores (the scale’s linearity) to better correspond to the increase in severity leading to higher risks of the studied outcome.

Our finding that the modified ROP-ActS is superior to grading ROP based on stage or zone alone is based on prediction analyses of the short-term outcome evaluating the initial progression of ROP up to the point of requiring treatment. The scale was not evaluated with respect to long-term outcome, although short-term outcomes are known to predict long-term outcomes (Hellstrom et al., 2018). The modified ROP-ActS should be further validated with regard to long-term outcome data. The ongoing multicentre randomized clinical trial (NCT04004208) administering Eylea to Type 1 ROP will explore the theoretical ROP activity scale as a secondary outcome (”).

In our study sample, the majority of infants (87.1%) had (as expected) no ROP diagnosed at the first visit, meaning that for most infants the first examinations (which took place according to the Swedish guidelines) were timely (Holmstrom et al., 2015). Yet, at the first examination, 24/535 (4.5%) infants had ROP diagnosed in zone II, stage 2 or 3; 10/24 (41.7%) had documented reasons as infant being very sick or difficult/incomplete examination, and 10/24 (41.7%) were treated for ROP after the initial examination.

While not studied in detail, there appears to be less variability of the postnatal age at first ROP treatment for the most immature infants, compared to greater variability for infants with higher GA at birth (data not shown). This may suggest that in the most immature infants, immaturity per se is the dominating risk factor for ROP treatment while for more mature infants, external factors may play a larger role in ROP development. Additionally, the progression from first ROP examination to first ROP treatment appears to be faster for extremely premature infants, suggesting a more rapid disease pattern with lower GA at birth.

Although the study cohort is relatively large for infants at high risk for ROP, only a few cases of very severe ROP were observed during the study period. Only two diagnoses of zone I disease were seen, one with stage 2 and one with stage 3. Moreover, in the study group there was no zone 1 with plus disease, nor any aggressive posterior ROP nor any stage 4a, 4b and 5 disease, resulting in an inability to evaluate these scores. However, the nine experts of the original article were in agreement regarding the severity of these most aggressive diagnoses so that validation of these extreme ends of the spectrum seems less urgent than for the intermediate scores where there was more disagreement among the nine expert graders of the original paper (Smith et al., 2019).

A strength of this study is that a standardized ROP protocol for data collection was used at the study site for each infant and ROP examination allowing statistical comparison of the observed ROP-ActS scores. Another strength is that data entry was complete in almost all cases with reason for missing data documented. The current study is representative of the complete Swedish cohort screened for ROP (Holmstrom et al., 2019).

As a limitation, the study is based on data from a single centre in Western Sweden offering advanced neonatal care and may not be generalizable to less developed countries, where timing of ROP treatment is often not based on theoretical guidelines, oxygen supplementation may not be monitored and infants with higher GA at birth are at risk for ROP (Zepeda-Romero & Gilbert, 2015). In the current study, all infants receiving ROP treatment were <28 weeks of GA at birth.

The modified ROP activity scale (ROP-ActS) shows better predictive ability for ROP treatment than ROP stage or zone alone. In addition, the modified scale’s sensitivity characteristic, studied through associations to GA, was better compared to ROP stage but not for ROP zone in our clinical cohort. Further evaluations on other populations and on long-term outcomes are recommended, including the scale’s most important and requested characteristic that is being a sensitive score when differentiating impact of provided preventative ROP treatments.

## Supplementary Material

1 **Appendix S1.** Logical partial order restrictions.
**Figure S1.** Distribution of original and modified ROP Activity Scale (ROP-ActS) over postnatal age by gestational age category.
**Figure S2.** Distribution of ROP stage over postnatal age by gestational age category.
**Figure S3.** Distribution of ROP zone over postnatal age by gestational age category.
**Table S1.** Original and modified ROP Activity Scale (ROP-ActS).

## Figures and Tables

**Table 1.** Descriptive data for study population overall and for infants with and without treatment.

Variable	All Infants (*n* = 535)	Not treated for ROP (*n* = 461)	Treated for ROP (*n* = 74)
Sex			
Boys	281 (52.5%)	239 (51.8%)	42 (56.8%)
Girls	254 (47.5%)	222 (48.2%)	32 (43.2%)
Birthweight (g)	1068 (358)	1129 (344)	689 (150)
	1020 (440; 2445)	1113 (450; 2445)	678 (440; 1005)
	*n* = 534	*n* = 460	*n* = 74
Birthweight SDS (for gestational age ≥ 24 weeks)	−1.16 (1.53)	−1.19 (1.57)	−0.842 (1.043)
	−0.86 (−8.42; 2.74)	−0.87 (−8.42; 2.74)	−0.724 (−3.665; 0.978)
	*n* = 500	*n* = 450	*n* = 50
Gestational age at birth (weeks)	27.8 (2.4)	28.3 (2.1)	24.6 (1.3)
	28.1 (22.4; 34.4)	28.7 (22.6; 34.4)	24.4 (22.4; 27.9)
Maximum ROP stage			
No ROP	297 (55.5%)	297 (64.4%)	0 (0.0%)
ROP stage 1	42 (7.9%)	42 (9.1%)	0 (0.0%)
ROP stage 2	89 (16.6%)	87 (18.9%)	2 (2.7%)
ROP stage 3	107 (20.0%)	35 (7.6%)	72 (97.3%)
Plus disease (among infants with diagnosed ROP)	60 (25.2%)		60 (81.1%)
Worst zone (among infants with diagnosed ROP)			
Zone I	2 (0.8%)	0 (0.0%)	2 (2.7%)
Zone II	166 (69.7%)	96 (58.5%)	70 (94.6%)
Zone III	70 (29.4%)	68 (41.5%)	2 (2.7%)

For categorical variables, n (%) are presented. For continuous variables, Mean (SD)/Median (Min; Max)/*n* = are presented. N is presented for variables having at least one missing value.

ROP = retinopathy of prematurity, SDS = standard deviation score.

**Table 2.** Evaluation of the prediction ability of original and modified ROP Activity Scale (ROP-ActS), ROP stage and zone on ROP treatment.

	Univariable logistic regression			Multivariable logistic regression adjusted for GA, BW, sex		Multivariable logistic regression mod. ROP-ActS and ROP stage		Multivariable logistic regression mod. ROP- ActS and ROP zone	
	OR (95% CI)	p-value	Area under ROC curve (95% CI)	OR (95% CI)	p-value	OR (95% CI)	p-value	OR (95% CI)	p-value
Maximum (most severe) value before 10 weeks of PNA^a^									
Original ROP-ActS (per 1 score increase)	1.52 (1.39–1.67)	<0.0001	0.81 (0.76–0.87)	1.39 (1.24–1.56)	<0.0001				
Modified ROP-ActS (per 1 score increase)	1.55 (1.41–1.71)	<0.0001	0.82 (0.76–0.87)	1.38 (1.23–1.55)	<0.0001	1.67 (1.35–2.10)	<0.0001	1.42 (1.06–1.91)	0.018
ROP stage(per 1 step increase in severity)	3.22 (2.42–4.27)	<0.0001	0.79 (0.73–0.84)	2.94 (1.98–4.37)	<0.0001	0.74 (0.36–1.53)	0.42		
ROP zone(per 1 step increase in severity)	4.56 (3.23–6.44)	<0.0001	0.81 (0.76–0.86)	2.99 (1.98–4.51)	<0.0001			1.39 (0.49–3.92)	0.54

BW = birthweight, GA = gestational age, OR = odds ratio, PNA = postnatal age, ROC = receiver operating characteristic, ROP = retinopathy of prematurity, ROP-ActS = ROP activity scale.

a Number of missing values is *n* = 11 due to either first visit occurring after 10 weeks of PNA or ROP treatment occurring before 10 weeks of PNA.

**Table 3.** Associations between gestational age and original and modified ROP Activity Scale (ROP-ActS), ROP stage and zone

	Univariable linear regression with GA as dependent variable^b^			Multivariable linear regression with GA as dependent variable^c^		Multivariable linear regression with GA as dependent variable^d^	
	Beta (95% CI)	p-value	*R* ^2^	Beta (95% CI)	p-value	Beta (95% CI)	p-value
Maximum (most severe) value before 10 weeks of PNA^a^							
All observations							
ROP-ActS	−0.42 (−0.48; −0.35)	<0.0001	0.23				
Mod. ROP-ActS	−0.42 (−0.48; −0.36)	<0.0001	0.26	−0.55 (−0.68; −0.42)	<0.0001	−0.31 (−0.52; −0.10)	0.0042
ROP stage	−1.08 (−1.29; −0.88)	<0.0001	0.17	0.45 (0.03; 0.87)	0.035		
ROP zone	−1.37 (−1.58; −1.17)	<0.0001	0.25			−0.40 (−1.09; 0.29)	0.26
All observations excluding no ROP							
ROP-ActS	−0.35 (−0.45; −0.24)	<0.0001	0.20				
Mod. ROP-ActS	−0.45 (−0.55; −0.34)	<0.0001	0.29	−0.53 (−0.64; −0.41)	<0.0001	−0.14 (−0.38; 0.10)	0.25
ROP stage	−0.32 (−0.85; 0.21)	0.23	0.01	0.75 (0.25; 1.24)	0.0032		
ROP zone	−2.51 (−3.06; −1.96)	<0.0001	0.32			−1.83 (−3.12; −0.53)	0.0059

ROP = retinopathy of prematurity; PNA = postnatal age; GA = gestational age; BW = birthweight; ROP-ActS = ROP activity scale

a Number of missing values is *n* = 11 due to either first visit occurring after 10 weeks of PNA or ROP treatment occurring before 10 weeks of PNA.

b All analyses are performed using univariable linear regression.

c Multivariable linear regression including mod. ROP-ActS and ROP stage. *R*^2^ = 0.26 for all observations, and *R*^2^ = 0.33 excluding no ROP.

d Multivariable linear regression including mod. ROP-ActS and ROP zone. *R*^2^ = 0.26 for all observations, and *R*^2^ = 0.32 excluding no ROP.
