# Supplementary material for: Validation of the Retinopathy of Prematurity Activity Scale (ROP-ActS) using retrospective clinical data
Source: Acta Ophthalmol. Author manuscript; Available in PMC 2021 Nov 27. (PMC8626862; doi:10.1111/aos.14532)
Supplement: 1 — Appendix S1. Logical partial order restrictions. Figure S1. Distribution of original and modified ROP Activity Scale (ROP-ActS) over postnatal age by gestational age category. Figure S2. Distribution of ROP stage over postnatal age by gestational age category. Figure S3. Distribution of ROP zone over postnatal age by gestational age category. Table S1. Original and modified ROP Activity Scale (ROP-ActS). [file NIHMS1756981-supplement-1.pdf]

Supplementary Online Content

Pivodic A, Nilsson S, Stahl A, Smith LEH, Hellström A. Validation of the Retinopathy of Prematurity Activity Scale (ROP-ActS) using retrospective clinical data

Appendix S1. Logical partial order restrictions

Figure S1. Distribution of original and modified ROP Activity Scale (ROP-ActS) over postnatal age by gestational age category.

Figure S2. Distribution of ROP stage over postnatal age by gestational age category.

Figure S3. Distribution of ROP zone over postnatal age by gestational age category.

Table S1. Original and modified ROP Activity Scale (ROP-ActS).

This supplementary material has been provided by the authors to give readers additional information about their work.

Appendix S1. Logical partial order restrictions

The logical partial order restrictions for the published ROP activity scale are based on the three parameters ordering characteristics expressing the severity of the retinopathy of prematurity (ROP) disease. One could think of a three-dimensional matrix for which in all three directions, moving from lower to higher matrix position in one direction when the other two directions are kept constant, the logical partial order is maintained if all the assigned scores in the matrix are increasing for increasing severity.

For example, we concluded that the published (first iteration) ROP activity scale is fulfilling this requirement, for which the three-dimensional matrix, here shown as two two-dimensional matrices, has the values as presented in the table below for scores 1-18.

**S(i,j,k)**	**No plus disease (i=1)**				**Plus disease (i=2)**		
	**Stage 1 (k=1)**	**Stage 2 (k=2)**	**Stage 3 (k=3)**		**Stage 1 (k=1)**	**Stage 2 (k=2)**	**Stage 3 (k=3)**
**Zone III (j=1)**	1	2	5		4	6	9
**Zone II (j=2)**	3	7	8		11	13	14
**Zone I (j=3)**	10	12	16		15	17	18

The orange cells are not observed and it is considered by the authors in this paper that are experts in this research area (A.S., L.E.H.S, A.H.) that those combinations are not clinically possible.

The gray cells are considered being possible but rare and not observed in the studied cohort.

The cells with the red colored font are those recommended to initiate ROP treatment according to the current guidelines.

Figure S1. Distribution of original and modified ROP Activity Scale (ROP-ActS) over postnatal age by gestational age category.

Mean and 95% CI are presented for the closest available value at or before certain postnatal week, implying that last value is carried forward until next visit.

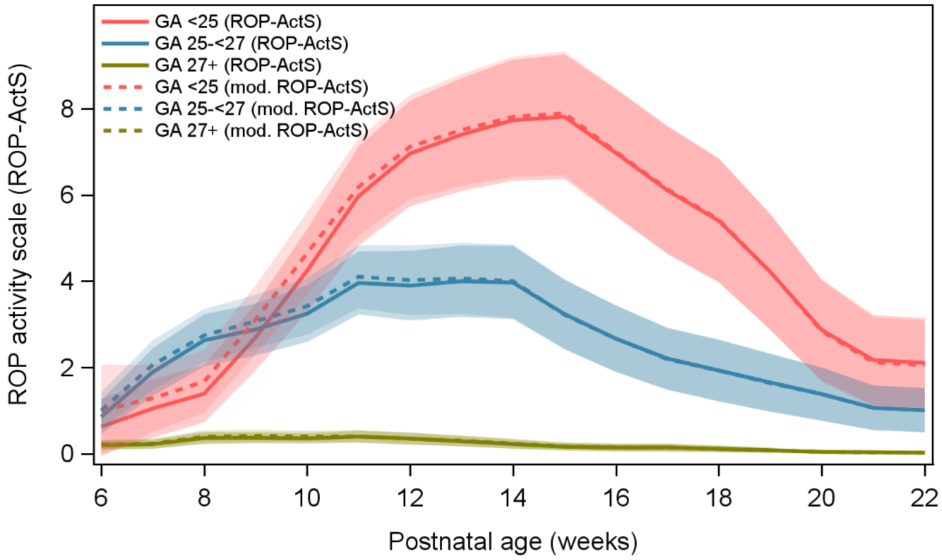

Figure S2. Distribution of ROP stage over postnatal age by gestational age category.

Percentage of infants with specific ROP stage are presented for the closest available value at or before certain postnatal week, implying that last value is carried forward until next visit.

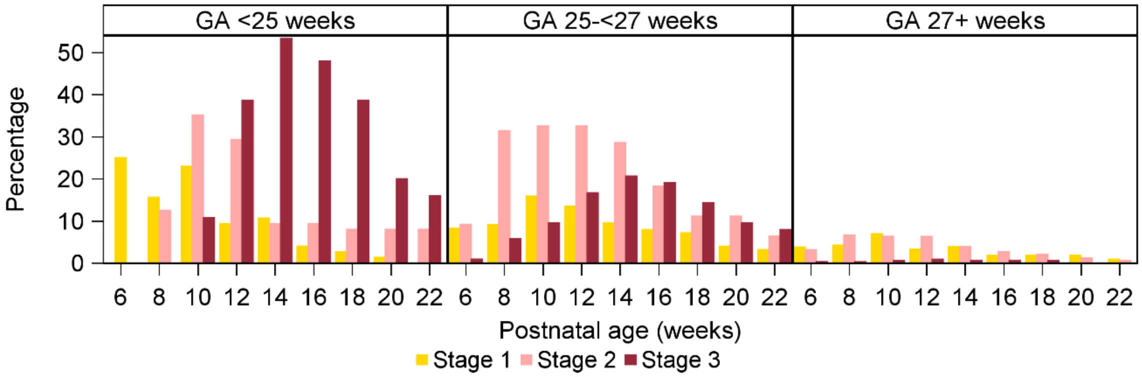

Figure S3. Distribution of ROP zone over postnatal age by gestational age category.

Percentage of infants with specific ROP zone are presented for the closest available value at or before certain postnatal week, implying that last value is carried forward until next visit.

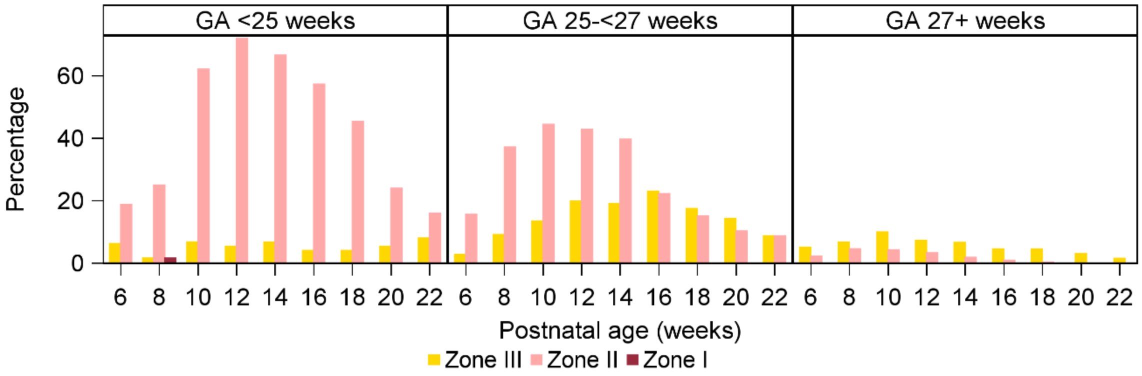

Table S1. Original and modified ROP Activity Scale (ROP-ActS)

Category [O]	Original ROP-ActS [O]	Zone	Stage	Plus disease	Aimed to be evaluated within the current study	Modified ROP-ActS [M]
Mild	0	None	None		Descriptively only	0
Mild	1	III	1		Yes	1
Mild	2	III	2		Yes	2
Mild	3	II	1		Yes	5
Mild	4	III	1	+	Yes (combination not clinically possible/observed)	*[4]*
Mild	5	III	3		Yes	3
Mild	6	III	2	+	Yes (combination not clinically possible/observed)	*[6]*
Mild	7	II	2		Yes	7
Moderate	8	II	3		Yes	8
Moderate	9	III	3	+	Yes	9
Moderate	10	I	1		Yes (combination not observed in the current cohort)	*[10]*
Moderate	11	II	1	+	Yes (combination not clinically possible/observed)	*[11]*
Moderate	12	I	2		Yes	12
Severe	13	II	2	+	Yes [ROPT]	13
Severe	14	II	3	+	Yes [ROPT]	14
Severe	15	I	1	+	Yes [ROPT] (combination not observed in the current cohort)	*[15]*
Severe	16	I	3		Yes [ROPT]	16
Severe	17	I	2	+	Yes [ROPT] (combination not observed in the current cohort)	*[17]*
Severe	18	I	3	+	Yes [ROPT] (combination not observed in the current cohort)	*[18]*
Severe	19	Any zone	AP-ROP		No – data not available for evaluation in this study	*[19]*
Severe	20	Any zone	4a		No – data not available for evaluation in this study	*[20]*
Severe	21	Any zone	4b		No – data not available for evaluation in this study	*[21]*
Severe	22	Any zone	5		No – data not available for evaluation in this study	*[22]*

[T] Original ROP activity scale (ROP-ActS) and categories (Smith et al, 2019)
[ROPT] Requires ROP treatment according to the Early Treatment of ROP criteria (ETROP, 2003)
[M] Modified ROP Activity Scale (ROP-ActS) proposed based on the risk evaluation regarding studied short-term outcome, the need for ROP treatment.
